# Chemical Shift Separated and Compensated Ultra‐Short Echo‐Time Imaging

**DOI:** 10.1002/mrm.70327

**Published:** 2026-02-27

**Authors:** Martin Krämer, Lumeng Cui, Jürgen R. Reichenbach, Stefan Sommer

**Affiliations:** ^1^ Institute of Diagnostic and Interventional Radiology, Jena University Hospital Friedrich Schiller University Jena Jena Germany; ^2^ Medical Physics Group, Institute of Diagnostic and Interventional Radiology Jena University Hospital, Friedrich Schiller University Jena Jena Germany; ^3^ Siemens Healthcare Limited Burnaby British Columbia Canada; ^4^ Swiss Center for Musculoskeletal Imaging (SCMI), Balgrist Campus Zurich Switzerland; ^5^ Swiss Innovation Hub Siemens Healthineers International AG Zurich Switzerland

**Keywords:** binomial pulses, chemical shift, Dixon, fat‐water separation, UTE

## Abstract

**Purpose:**

To develop a modified binomial excitation scheme for ultra‐short echo‐time (UTE) imaging that allows for the separation of water and fat signals and the correction of chemical shift artifacts.

**Methods:**

Theoretical derivation and numerical Bloch simulations were performed for a modified water‐excitation binomial pulse scheme to calculate fat and water signals as well as a chemical shift corrected UTE image from two acquisitions with these configurations. Chemical shift correction was applied at the readout level by multiplying the fat signal with a corresponding linear phase term. Measurements with the proposed binomial pulse scheme were performed at 3 T and 7 T.

**Results:**

Imaging results at 7 T and 3 T showed robust fat and water separation and images free of chemical shift‐induced blurring after applying the proposed method. Compared to standard UTE, the corrected UTE images exhibited improved tissue boundary delineation and improved visibility of fine details. Bloch simulations demonstrated that deviations of the phase differences between water and fat from the analytical solution were below 1% for typical UTE acquisition parameters.

**Conclusion:**

The proposed binomial excitation scheme and post‐processing effectively separate water and fat signals and correct for chemical shift artifacts in UTE imaging, providing improved image quality, especially at high field strengths.

## 1 Introduction

Chemical shift in MRI refers to the phenomenon where different nuclei in a molecule resonate at slightly different frequencies due to their distinct chemical environments (e.g., fat having a different resonance frequency from water). Consequently, fat spins will accumulate an additional time‐dependent phase offset during readout that shifts their spatial location in the reconstructed image. This is typically a problem across the readout direction (frequency encoding) [1], but can also occur in slice encoding direction [2]. Appearing as dark or bright lines, the resulting artifacts, typically at the border between water and fat, can obscure important anatomical details and significantly impact diagnostic quality and reliability [3].

This problem becomes more pronounced for non‐Cartesian sequences, where the chemically shifted compartments are displaced in all encoding directions rather than only along a single readout direction. In 3D ultra‐short echo‐time (UTE) imaging that uses a 3D center‐out radial trajectory [4, 5], the chemical shift effectively blurs and obstructs fat/water boundaries in all spatial directions [6]. Imaging at high field strengths further amplifies the problem, since the chemical shift is proportional to the main magnetic field [6]. As a result, chemical shift related artifacts are much more pronounced [7, 8].

Several strategies have been developed to mitigate the effects of chemical shift in MRI. One approach is to adjust the image acquisition parameters [3]. For example, swapping the phase‐ and frequency‐encoding directions (for Cartesian imaging) can rotate the signal superposition resulting from chemical shift differences to a less compromising image area. Increasing the receiver bandwidth (at the cost of signal‐to‐noise ratio (SNR)) also helps by allowing each pixel to represent a greater frequency range, thereby reducing (but not eliminating) chemical shift induced displacement.

Another effective solution is to apply fat suppression techniques, which aim to eliminate the fat signal, thus preventing chemical shift artifacts. However, these methods typically come at the cost of an increased total acquisition time and might fail in regions of increased field inhomogeneity. When combining inversion‐based fat suppression techniques with UTE imaging, the acquisition time can become highly inefficient [9, 10] due to the prolonged repetition times (TR) as UTE sequences typically require short TR for time‐efficient 3D sampling. Spectral fat saturation methods [11] are similarly challenging, as the frequency‐selective saturation pulse typically contributes to longer acquisition times of the actual UTE acquisition. This is often counterbalanced by acquiring several readout spokes per fat saturation preparation pulse, which, however, can lead to a partial suppression of fat. Furthermore, frequency‐selective saturation pulses can compromise very short‐T_2_ signals by saturating part of their broad spectra [12].

A different approach is to apply Dixon‐type [13, 14] sequences, which acquire multiple echoes at different echo times (TE) to enable separation of water and fat. Dixon sequences provide robust fat‐water separation; however, they involve longer scan times, complex post‐processing, and potential sensitivity to motion or field inhomogeneities. Furthermore, when combined with a UTE acquisition, the second echo is no longer acquired at an UTE.

Alternatively, water excitation (WE) techniques, such as binomial pulses, selectively excite water protons in MRI [15, 16, 17] to achieve a robust fat suppression, enhancing tissue contrast and minimizing chemical shift artifacts. Binomial pulse sequences, including 1‐1, 1‐2‐1, or 1‐3‐3‐1 configurations, selectively excite water protons by leveraging the in‐phase and out‐of‐phase timings caused by the resonance frequency difference between fat and water, offering compatibility with a wide range of MRI systems. These methods are particularly effective at high magnetic fields, where the reduced out‐of‐phase time enhances time efficiency due to shorter inter‐pulse delays. However, these techniques remain sensitive to magnetic field inhomogeneities, which may compromise fat suppression or necessitate precise timing or accurate shimming.

In this work, we propose a modified binomial excitation that allows for a separate reconstruction of water and fat as well as chemical shift‐corrected UTE images.

## 2 Theory

For solving the Bloch equations in the presence of a binomial pulse of the most basic form 1‐1 (two identical short rectangular pulses with equal amplitude), we define $\vec{M}\left(0\right)=\left(0,0,M_{0}\right)$ and $B_{1}=\alpha /\left(\gamma \tau \right)$ as initial conditions, where α and τ denote the flip angle and pulse duration of each pulse, respectively. For simplicity, we neglect off‐resonance or chemical shift during the RF‐pulse, that is, $\Delta B_{0}=0$, and only take it into account in the time interval between the two pulses. It will be shown later through numerical Bloch simulations that this is a justified approximation for reasonable chemical shifts and sufficiently short pulses. Additionally, relaxation during the short pulses is also neglected. The following considerations assume a rotating frame of reference for the on‐resonant component and a second off‐resonant component. For simplicity, we assume those to be water and fat, respectively. We further approximate fat as a single spectral peak corresponding to the dominant methylene component, allowing for the following analytical description of the phase evolution relative to the on‐resonant water component.

After a single RF‐pulse $RF_{1}$ with no phase‐offset (0°), the magnetization at the end of the RF‐pulse is given for both fat and water as follows:

(1) $$\vec{M}\left(\tau \right)=M_{W/F}\;\left(0,-\sin\alpha ,\cos\alpha \right).$$

**FIGURE 1:**
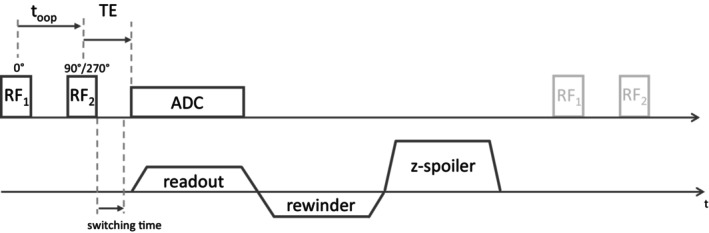
Schematic representation of a 3D UTE center‐out imaging sequence with added binomial excitation scheme using two short rectangular RF‐pulses $RF_{1}$ and $RF_{2}$ that are separated by the fat and water opposed‐phase time $t_{\mathrm{oop}}$. The second $RF_{2}$ pulse is applied with a phase of either 90° or 270° to acquire the respective first and second sequence repetition. Following $RF_{2}$, the readout starts immediately at the center of k‐space after a brief switching delay. Gradient spoiling is applied along the z‐direction after a balanced rewinder returns the trajectory back to the center of k‐space, ensuring a constant spoiling moment independent of the readout direction.

In the proposed binomial 1‐1 pulse scheme (Figure 1), the second pulse $RF_{2}$ has the same amplitude as $RF_{1}$ and is applied after a time delay of $t_{\mathrm{oop}}$, given as $t_{\mathrm{oop}}=\frac{1}{2\Delta f}$, with Δf being the off‐resonance frequency of fat in hertz. In the case of water, this leads to no change of the magnetization after $t_{\mathrm{oop}}$, while the magnetization vector of fat rotates by 180°. Since $t_{\mathrm{oop}}$ is much larger than the pulse duration, we take $T_{2}^{*}$‐decay into account during the delay time between the RF‐pulses, resulting in the respective magnetization vectors before the second RF‐pulse:

(2) $${\vec{M}}_{W}\left(\tau +t_{\mathrm{oop}}\right)=M_{W}\;\left(0,-\sin\alpha \cdot e^{-t_{oop}/T_{2,W}^{*}},\cos\alpha \right),$$

(3) $${\vec{M}}_{F}\left(\tau +t_{\mathrm{oop}}\right)=M_{F}\;\left(0,\sin\alpha \cdot e^{-t_{\mathrm{oop}}/T_{2,F}^{*}},\cos\alpha \right).$$

If the second RF‐pulse $RF_{2}$ is played out with a phase of 90° with respect to the first pulse, that is, ${\mathrm{RF}}_{1}^{0{}^{\circ}}-{\mathrm{RF}}_{2}^{90{}^{\circ}}$, the resulting magnetizations at the end of the second RF‐pulse become:

(4) $${\vec{M}}_{W,90}\left(\tau +t_{\mathrm{oop}}+\tau \right)=M_{W}\;\left(\cos\alpha \sin\alpha ,-\sin\alpha \cdot e^{-t_{oop}/T_{2,W}^{*}},\cos^{2}\alpha \right),$$

(5) $${\vec{M}}_{F,90}\left(\tau +t_{\mathrm{oop}}+\tau \right)=M_{F}\;\left(\cos\alpha \sin\alpha ,\sin\alpha \cdot e^{-t_{\mathrm{oop}}/T_{2,F}^{*}},\cos^{2}\alpha \right).$$

If instead the second pulse uses a phase of 270° with respect to the first pulse, that is, ${\mathrm{RF}}_{1}^{0{}^{\circ}}-{\mathrm{RF}}_{2}^{270{}^{\circ}}$, the resulting magnetizations will be:

(6) $${\vec{M}}_{W,270}\left(\tau +t_{\mathrm{oop}}+\tau \right)=M_{W}\;\left(-\cos\alpha \sin\alpha ,-\sin\alpha \cdot e^{-t_{oop}/T_{2,W}^{*}},\cos^{2}\alpha \right),$$

(7) $${\vec{M}}_{F,270}\left(\tau +t_{\mathrm{oop}}+\tau \right)=M_{F}\;\left(-\cos\alpha \sin\alpha ,\sin\alpha \cdot e^{-t_{\mathrm{oop}}/T_{2,F}^{*}},\cos^{2}\alpha \right).$$

The magnitude of the transverse magnetization is identical for water and fat in both binomial pulse configurations given by:

(8) $$|{\vec{M}}_{W,\mathrm{bp}}^{\mathrm{xy}}|=|{\vec{M}}_{W,90}^{\mathrm{xy}}|=|{\vec{M}}_{W,270}^{\mathrm{xy}}|=M_{W}\sin\left(\alpha \right)\sqrt{e^{-2t_{oop}⁄T_{2,W}^{*}}+\cos^{2}\alpha },$$

(9) $$|{\vec{M}}_{F,\mathrm{bp}}^{\mathrm{xy}}|=|{\vec{M}}_{F,90}^{\mathrm{xy}}|=|{\vec{M}}_{F,270}^{\mathrm{xy}}|=M_{F}\sin\left(\alpha \right)\sqrt{e^{-2t_{\mathrm{oop}}⁄T_{2,F}^{*}}+\cos^{2}\alpha }.$$

Considering two acquisitions with both configurations (${\mathrm{RF}}_{1}^{0{}^{\circ}}-{\mathrm{RF}}_{2}^{90{}^{\circ}}$ and ${\mathrm{RF}}_{1}^{0{}^{\circ}}-{\mathrm{RF}}_{2}^{270{}^{\circ}}$), the calculation of the phase difference between the respective transverse magnetizations for water and fat leads to

(10) $$\begin{array}{ll} \Delta \phi_{W} & =\arg\left(\frac{{\vec{M}}_{W,270}^{\mathrm{xy}}}{{\vec{M}}_{W,90}^{\mathrm{xy}}}\right)\\ & =\arg\left(\frac{-\cos^{2}\alpha -2i\cos\alpha \cdot e^{-t_{\mathrm{oop}}/T_{2,W}^{*}}+e^{-2t_{\mathrm{oop}}/T_{2,W}^{*}}}{\cos^{2}\alpha +e^{-2t_{\mathrm{oop}}/T_{2,W}^{*}}}\right), \end{array}$$

(11) $$\begin{array}{ll} \Delta \phi_{F} & =\arg\left(\frac{{\vec{M}}_{F,270}^{\mathrm{xy}}}{{\vec{M}}_{F,90}^{\mathrm{xy}}}\right)\\ & =\arg\left(\frac{-\cos^{2}\alpha +2i\cos\alpha \cdot e^{-t_{\mathrm{oop}}/T_{2,F}^{*}}+e^{-2t_{\mathrm{oop}}/T_{2,F}^{*}}}{\cos^{2}\alpha +e^{-2t_{\mathrm{oop}}/T_{2,F}^{*}}}\right). \end{array}$$

Because $\cos^{2}\alpha +e^{-2t_{\mathrm{oop}}/T_{2,W/F}^{*}}$ is always real and positive, these expressions can be simplified to

(12) $$\Delta \phi_{W}=\arg\left(-\cos^{2}\alpha -2i\cos\alpha \cdot e^{-t_{\mathrm{oop}}/T_{2,W}^{*}}+e^{-2t_{\mathrm{oop}}/T_{2,W}^{*}}\right),$$

(13) $$\Delta \phi_{F}=\arg\left(-\cos^{2}\alpha +2i\cos\alpha \cdot e^{-t_{\mathrm{oop}}/T_{2,F}^{*}}+e^{-2t_{\mathrm{oop}}/T_{2,F}^{*}}\right),$$

where for α in [0,π/2], the results lead to $\Delta \phi_{W}<0$ and $\Delta \phi_{F}>0$.

**FIGURE 2:**
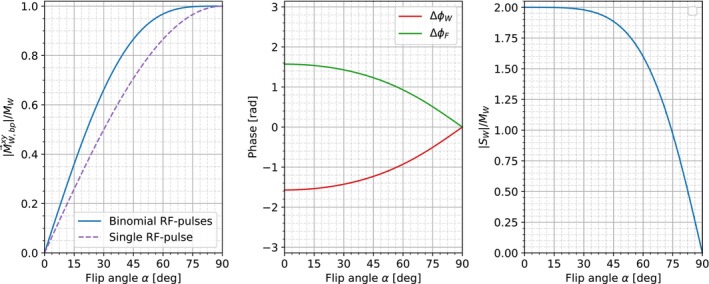
Left: Dependence of the transverse magnetization on the flip angle for a conventional single RF‐pulse (dashed purple line) compared to the proposed binomial pulse scheme (blue line). Middle: Phase differences of water $\Delta \Phi_{W}$ (red) and fat $\Delta \Phi_{F}$ (green) between the two binomial pulse configurations as a function of the flip angle. Right: Magnitude of the obtained water signal as a function of the flip angle. The curves on the left and right graphs are shown only for water since the magnitude of the fat signal is identical. All three graphs assume $T_{2}^{*}>>t_{\mathrm{oop}}$.

For $T_{2}^{*}>>t_{\mathrm{oop}}$ the phase difference between ${\vec{M}}_{270}^{\mathrm{xy}}$ and ${\vec{M}}_{90}^{\mathrm{xy}}$ for water and fat approach ∓π/2 for α→0 and 0 for α→π/2 (Figure 2). Because they describe the phase difference of the respective fat and water magnetization vectors, we can relate the water and fat magnetizations from ${\mathrm{RF}}_{1}^{0{}^{\circ}}-{\mathrm{RF}}_{2}^{90{}^{\circ}}$ and ${\mathrm{RF}}_{1}^{0{}^{\circ}}-{\mathrm{RF}}_{2}^{270{}^{\circ}}$ by:

(14) $${\vec{M}}_{W,270}^{\mathrm{xy}}={\vec{M}}_{W,90}^{\mathrm{xy}}e^{i\Delta \phi_{W}},$$

(15) $${\vec{M}}_{F,270}^{\mathrm{xy}}={\vec{M}}_{F,90}^{\mathrm{xy}}e^{i\Delta \phi_{F}}.$$

After defining the measured signals as the sum of the water and fat components

(16) $${\vec{S}}_{90}={\vec{M}}_{W,90}^{\mathrm{xy}}+{\vec{M}}_{F,90}^{\mathrm{xy}},$$

(17) $${\vec{S}}_{270}={\vec{M}}_{W,270}^{\mathrm{xy}}+{\vec{M}}_{F,270}^{\mathrm{xy}},$$

we can calculate a signal that contains only water $S_{W}$ contributions as follows:

(18) $${\vec{S}}_{W}={\vec{S}}_{270}e^{i\left(\pi -\Delta \phi_{F}\right)}+{\vec{S}}_{90},$$

(19) $${\vec{S}}_{W}=\left({\vec{M}}_{W,270}^{\mathrm{xy}}+{\vec{M}}_{F,270}^{\mathrm{xy}}\right)e^{i\left(\pi -\Delta \phi_{F}\right)}+{\vec{M}}_{W,90}^{\mathrm{xy}}+{\vec{M}}_{F,90}^{\mathrm{xy}},$$

(20) $${\vec{S}}_{W}=\left({{\vec{M}}_{W,90}^{\mathrm{xy}}e^{i\Delta \phi_{W}}+\vec{M}}_{F,90}^{\mathrm{xy}}e^{i\Delta \phi_{F}}\;\right)e^{i\left(\pi -\Delta \phi_{F}\right)}+{\vec{M}}_{W,90}^{\mathrm{xy}}+{\vec{M}}_{F,90}^{\mathrm{xy}},$$

(21) $${\vec{S}}_{W}={\vec{M}}_{W,90}^{\mathrm{xy}}\left(1+e^{i\left(\pi +\Delta \phi_{W}-\Delta \phi_{F}\right)}\right),$$

with

(22) $${\vec{M}}_{F,90}^{\mathrm{xy}}e^{i\Delta \phi_{F}}\cdot e^{i\left(\pi -\Delta \phi_{F}\right)}={\vec{M}}_{F,90}^{\mathrm{xy}}e^{i\pi }=-{\vec{M}}_{F,90}^{\mathrm{xy}}.$$

To obtain a signal that contains only fat ${\vec{S}}_{F}$, the same can be done using $\pi +{\Delta \phi }_{W}$ applied to ${\vec{S}}_{90}$ instead:

(23) $${\vec{S}}_{F}={\vec{S}}_{90}e^{i\left(\pi +\Delta \phi_{W}\right)}+{\vec{S}}_{270},$$

(24) $${\vec{S}}_{F}={\vec{M}}_{F,270}^{\mathrm{xy}}\left(1+e^{i\left(\pi +\Delta \phi_{W}-\Delta \phi_{F}\right)}\right).$$

**FIGURE 3:**
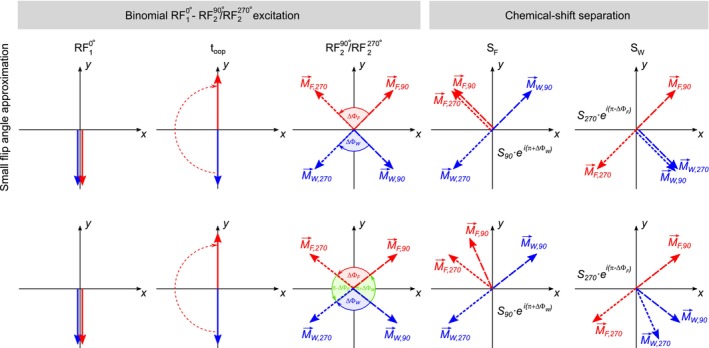
Orientation of the water (blue) and fat (red) magnetization vectors after applying the proposed binomial pulse scheme (first three columns) and after applying the respective phase offsets to $S_{90}$ and $S_{270}$ (last two columns) during post‐processing. The top row illustrates the ideal condition for small flip angles that leads to a perfect constructive/destructive overlap of the fat and water magnetizations. The bottom row shows the case for larger flip angles that leads to only a perfect cancellation of the water (fourth column) and fat (last column) with slightly imperfect overlap of the respective other components.

The orientation of the magnetization vectors before and after applying the respective phase offsets to ${\vec{S}}_{90}$ and ${\vec{S}}_{270}$ is visualized as a vector model in Figure 3.

When ignoring $T_{2}^{*}$ decay during $t_{\mathrm{oop}}$, that is, $T_{2}^{*}>>t_{\mathrm{oop}}$, we can relate $\Delta \phi_{W}=-\Delta \phi_{F}$ and rewrite these as follow:

(25) $${\vec{S}}_{W}={\vec{M}}_{W,90}^{\mathrm{xy}}\left(1+e^{i\left(\pi -2\Delta \phi_{F}\right)}\right)={2\cdot \vec{M}}_{W,90}^{\mathrm{xy}}\sin\Delta \phi_{F}\cdot e^{i\left(\pi /2-\Delta \Phi_{F}\right)},$$

(26) $${\vec{S}}_{F}={\vec{M}}_{F,270}^{\mathrm{xy}}\left(1+e^{i\left(\pi -2\Delta \phi_{F}\right)}\right)={-2\cdot \vec{M}}_{F,270}^{\mathrm{xy}}\sin\Delta \phi_{F}\cdot e^{i\left(\pi /2-\Delta \Phi_{F}\right)},$$

with respective magnitudes of

(27) $$|S_{W}|=2\cdot M_{W}\cdot \left|\sin\Delta \phi_{F}\right|,$$

(28) $$|S_{F}|=2\cdot M_{F}\cdot \left|\sin\Delta \phi_{F}\right|.$$

These equations show that for $T_{2}^{*}>>t_{\mathrm{oop}}$ and small flip angles one obtains twice the water and fat magnetizations (Figure 2), which is the optimal case when combining two separate acquisitions. The small‐angle approximation is crucial for the linear combination of the two signals, as it ensures the system operates in a linear regime by preventing significant $M_{z}$ saturation and non‐linear coupling effects.

## 3 Methods

### 3.1 Bloch Simulations

One limitation of the equations derived in the theory section is the assumption of ignoring off‐resonance effects during the RF‐pulse. For longer pulse durations or large off‐resonance frequencies, the off‐resonant spins will acquire an additional phase offset during the RF‐pulse, potentially leading to deviations from the derived $\Delta \phi_{W}$ and $\Delta \phi_{F}$ (Equations 12 and 13). To investigate the influence of off‐resonance during the RF‐pulses on $\Delta \phi_{W}$ and $\Delta \phi_{F}$, numerical Bloch simulations were performed using an in‐house developed Python (version 3.10, Python Software Foundation, https://www.python.org/) framework with the NumPy library (version 2.2). For simplicity, only RF‐interactions with two ensembles of fat and water spins were simulated without any gradient activity. *T*_1_ and *T*_2_ relaxation were neglected, and only a single repetition of the sequence was modeled, using a discretization interval of 0.001 μs. Simulations were performed with off‐resonance frequencies of 0, 440, and 1040 Hz, corresponding to no chemical shift and to the 3.5 ppm chemical shift of fat at field strength of 3 T and 7 T, respectively.

### 3.2 Fat/Water Decomposition and Chemical‐Shift Correction

From the two acquisitions with binomial pulse schemes ${\mathrm{RF}}_{1}^{0{}^{\circ}}-{\mathrm{RF}}_{2}^{90{}^{\circ}}$ and ${\mathrm{RF}}_{1}^{0{}^{\circ}}-{\mathrm{RF}}_{2}^{270{}^{\circ}}$, the respective fat and water signals $S_{F}$ and $S_{W}$ were calculated according to Equations (23) and (18). Specifically, these signals were obtained by multiplying measured signal $S_{90}$ and $S_{270}$ with respective phase shifts proportional to $\pi -\Delta \phi_{F}$ and $\pi +\Delta \phi_{W}$, followed by an addition of the two terms. Although this procedure can be done on the reconstructed images $I_{270}$ and $I_{90}$, we performed it directly on the signal/readout level, before any reconstruction or post‐processing step while neglecting $T_{2}^{*}$ relaxation and applying simplified phase shifts of

(29) $$\Delta \phi_{W}=\arg\left(-\cos^{2}\left(\alpha \right)-2i\cos\left(\alpha \right)+1\right),$$

(30) $$\Delta \phi_{F}=\arg\left(-\cos^{2}\left(\alpha \right)+2i\cos\left(\alpha \right)+1\right).$$

Applying the fat/water decomposition at the readout level is possible because the signals from both binomial pulse configurations are acquired using identical readout parameters, including the echo‐time. By operating at the readout level, the chemical shift along the readout of $S_{F}$ can be corrected by multiplying $S_{F}$ with a corresponding linear phase ramp of

(31) $$S_{F,\text{corr}}^{n}=S_{F}^{n}\cdot e^{i2\pi \cdot \Delta t\cdot n\cdot \Delta f},$$

with Δt being the dwell time and n the *n‐*th readout sampling point. After calculating an off‐resonance corrected fat signal, the corresponding non‐fat saturated UTE signal, free of chemical shift displacement artifacts, was calculated as follows:

(32) $$S_{\text{corr}}=S_{w}+S_{F,\text{corr}}.$$

### 3.3 MRI Acquisition

The proposed binomial excitation scheme was implemented in a UTE research application sequence. A schematic pulse diagram is shown in Figure 1. To account for deviations from the nominal trajectory caused by gradient system imperfections, a Gradient Impulse Response Function (GIRF) correction [18] based on a vendor supplied calibration was applied to all scans during reconstruction. All scans used ramp sampling and a readout oversampling factor of 2. Three healthy subjects were scanned on a 3 T (MAGNETOM Prisma, Siemens Healthineers, Forchheim, Germany) and on a 7 T (MAGNETOM Terra.X, Siemens Healthineers, Forchheim, Germany) scanner. All volunteers gave written informed consent according to the guidelines of the local ethics committees.

High‐resolution knee imaging at 7 T was performed using a vendor‐supplied knee coil (1Tx28Rx knee coil, Quality Electrodynamics QED, Mayfield, OH) with acquisition parameters: (176 × 117 × 92) mm^3^ field‐of‐view, (0.5 × 0.5 × 1.25) mm^3^ voxel size, 3D radial center‐out trajectory, 384 sampling points per readout, τ= 40 μs, $t_{\mathrm{oop}}=0.5\;\mathrm{ms}$, $\alpha =3^{{}^{\circ}}$, 70 μs TE, 4 ms TR, 4.8 μs dwell‐time, 106 676 radial spokes and a 7.1 min acquisition time (TA) for one scan. Anisotropic resolution and field‐of‐view were achieved by scaling the readout gradients to create an ellipsoidal k‐space coverage, while modulating the polar and azimuthal angular increments to ensure uniform sampling density across the ellipsoid surface. This scan was repeated three times using the ${\mathrm{RF}}_{1}^{0{}^{\circ}}-{\mathrm{RF}}_{2}^{90{}^{\circ}}$ and ${\mathrm{RF}}_{1}^{0{}^{\circ}}-{\mathrm{RF}}_{2}^{270{}^{\circ}}$ pulse configurations as well as a single pulse acquisition serving as a reference scan for standard UTE imaging. The applied delay time $t_{\mathrm{oop}}$ of 0.5 ms corresponds to a chemical shift of 993 Hz, which was manually estimated after shimming and frequency adjustment by measuring the spectral peak separation between water and fat in the vendor's frequency adjustment interface prior to the scan. This manually measured chemical shift was also used for the slope of the phase correction in the chemical shift compensation (Equation 31).

Imaging at a clinical field strength of 3 T was performed using a vendor‐supplied 16‐channel foot/ankle coil with the following acquisition parameters: (160 × 160 × 160) mm^3^ field‐of‐view, (0.6 × 0.6 × 0.6) mm^3^ voxel size, 3D radial center‐out trajectory, 256 sampling points per readout, pulse duration τ=40μs, $t_{\mathrm{oop}}=1.19\;\mathrm{ms}$ (corresponding to a chemical shift of 420 Hz), $\alpha =5^{{}^{\circ}}$, 40 μs TE, 5 ms TR, 3.2 μs dwell‐time, 60 000 radial views and a 10.2 min total acquisition time (TA). In contrast to the 7 T scan, the acquisitions of ${\mathrm{RF}}_{1}^{0{}^{\circ}}-{\mathrm{RF}}_{2}^{90{}^{\circ}}$ and ${\mathrm{RF}}_{1}^{0{}^{\circ}}-{\mathrm{RF}}_{2}^{270{}^{\circ}}$ were acquired in an interleaved fashion acquiring the same readout spoke direction with both sets of pulses one after the other.

Additional imaging of a volunteer's hand and wrist was performed at 3 T using two 8 channel Noras Variety Flex coils (NORAS MRI products GmbH, Höchberg, Germany) with the following acquisition parameters: (200 × 100 × 60) mm^3^ field‐of‐view, (0.6 × 0.6 × 0.6) mm^3^ voxel size, 3D radial center‐out trajectory, 362 sampling points per readout, τ=40μs, $t_{\mathrm{oop}}=1.19\;\mathrm{ms}$, $\alpha =4^{{}^{\circ}}$, 100 μs TE, 4.3 ms TR, 5.2 μs dwell‐time, 97 424 radial spokes and a 7.0 min acquisition time (TA) for one scan, which was repeated 3 times using the ${\mathrm{RF}}_{1}^{0{}^{\circ}}-{\mathrm{RF}}_{2}^{90{}^{\circ}}$ and ${\mathrm{RF}}_{1}^{0{}^{\circ}}-{\mathrm{RF}}_{2}^{270{}^{\circ}}$ pulse configurations as well as a single pulse acquisition serving as a reference scan for standard UTE imaging.

Phantom measurements were performed to investigate the robustness of the proposed method against static magnetic field inhomogeneities. Measurements were performed on a fat‐water phantom consisting of two bottles containing vegetable oil and gadolinium‐doped water, respectively. The phantom was scanned at 3 T using a vendor‐supplied single‐channel Tx/Rx knee coil with the following acquisition parameters: (220 × 220 × 220) mm^3^ field‐of‐view, (2.0 × 2.0 × 2.0) mm^3^ voxel size, 3D radial center‐out trajectory, 140 sampling points per readout, τ=40μs, $t_{\mathrm{oop}}=1.19\;\mathrm{ms}$ (corresponding to a chemical shift of 420 Hz), $\alpha =4^{{}^{\circ}}$, 100 μs TE, 4 ms TR, 8.2 μs dwell‐time and 39 496 radial views. To simulate varying degrees of field inhomogeneity, the scans were performed under three conditions: (1) optimal shim after automatic shimming by the scanner, (2) an induced linear gradient offset of +25 μT/m along the z‐direction, and (3) an induced linear gradient offset of +50 μT/m along the z‐direction. For comparison, standard binomial water ${\mathrm{RF}}_{1}^{0{}^{\circ}}-{\mathrm{RF}}_{2}^{0{}^{\circ}}$ and fat excitation ${\mathrm{RF}}_{1}^{0{}^{\circ}}-{\mathrm{RF}}_{2}^{180{}^{\circ}}$scans were acquired in addition to the proposed ${\mathrm{RF}}_{1}^{0{}^{\circ}}-{\mathrm{RF}}_{2}^{90{}^{\circ}}$and ${\mathrm{RF}}_{1}^{0{}^{\circ}}-{\mathrm{RF}}_{2}^{270{}^{\circ}}$scans for all shim configurations. Reference $\Delta B_{0}$ maps were calculated using the same sequence in a conventional single‐pulse excitation configuration by acquiring a second echo at 2.4 ms and estimating the field map from the phase difference between the two echoes.

## 4 Results

### 4.1 Bloch Simulations

**FIGURE 4:**
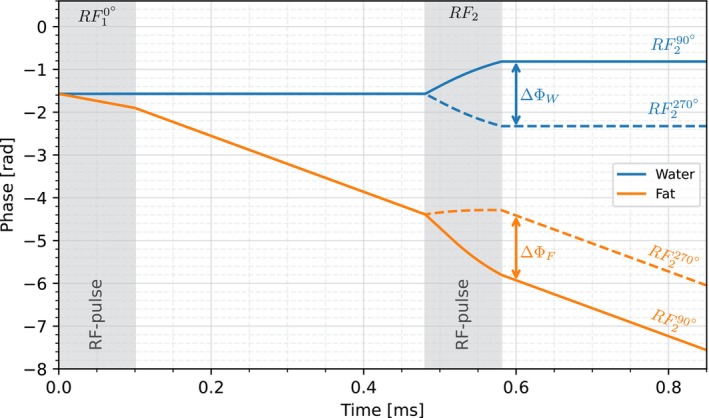
Phase evolution of the transverse magnetization as a function of time for the binomial pulse configurations ${\mathrm{RF}}_{1}^{0{}^{\circ}}-{\mathrm{RF}}_{2}^{90{}^{\circ}}$ and ${\mathrm{RF}}_{1}^{0{}^{\circ}}-{\mathrm{RF}}_{2}^{270{}^{\circ}}$. Shown in blue is the water signal and in orange the fat signal for an off‐resonance frequency of 1040 Hz, corresponding to the 3.5 ppm chemical shift of fat at a field strength of 7 T. Shaded gray areas represent the periods during which RF‐pulses of 100 μs duration were active. Simulations were performed with a pulse flip angle of 20°.

**FIGURE 5:**
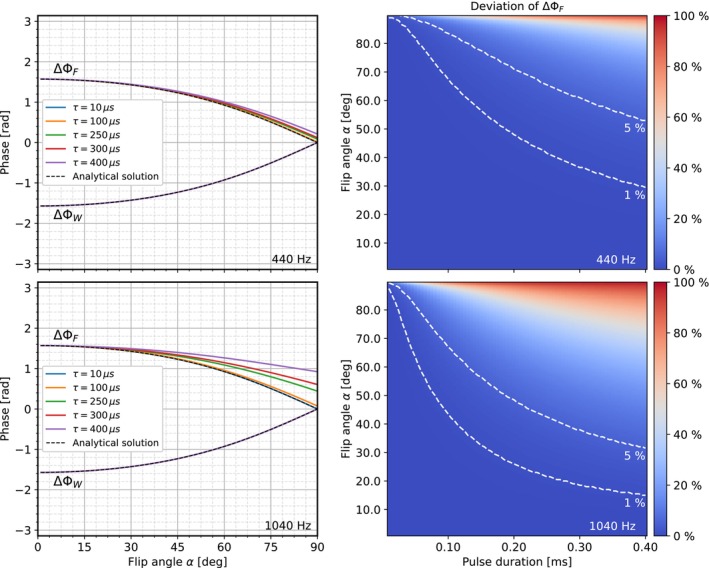
Deviation of $\Delta \Phi_{W}$ and $\Delta \Phi_{F}$ from the theoretical values derived from the equations in the theory section for flip angles from 0° to 90° and RF‐pulse durations from 10 to 400 μs. Overlaid on the right graphs in white are the contour lines that indicate deviations of $\Delta \Phi_{F}$ from theoretical prediction of 1% and 5%, respectively. The top row shows the results for an off‐resonance frequency of 440 Hz (fat at 3 T) and the bottom row for 1040 Hz (fat at 7 T).

Simulations of the phase evolution as a function of time show how the water and fat phases converge for the ${\mathrm{RF}}_{1}^{0{}^{\circ}}-{\mathrm{RF}}_{2}^{90{}^{\circ}}$ binomial pulse configuration and diverge for the ${\mathrm{RF}}_{1}^{0{}^{\circ}}-{\mathrm{RF}}_{2}^{270{}^{\circ}}$ counterpart (Figure 4) during application of the respective second pulse. When no pulse is active, the water signal exhibits no phase evolution due to being on‐resonant, whereas the fat signal shows a constant phase evolution corresponding to its chemical shift. In the simulation, the respective phase differences $\Delta \Phi_{W}$ and $\Delta \Phi_{F}$ (Figure 5, left) approach the predicted limits of ∓π/2 for α→0and 0 for α→π/2, with increasing deviation of $\Delta \phi_{F}$ observed for longer pulse durations and higher flip angles. $\Delta \phi_{W}$ on the contrary shows no deviation from the theoretical approximation. The deviation of $\Delta \phi_{F}$ from the analytical solution remains below 1% for short pulse durations and small flip angles (Figure 5, right). The threshold at which the deviation from the theory exceeds 1% shifts toward shorter pulses and smaller flip angles with increasing off‐resonance frequency. Nevertheless, even at an off‐resonance frequency of 1040 Hz (corresponding to the fat chemical shift at 7 T), the deviation remains well below 1% for reasonable flip angles (< 20°) and pulse durations (< 200 μs) typically used in UTE‐based acquisitions.

### 4.2 Imaging Results

**FIGURE 6:**
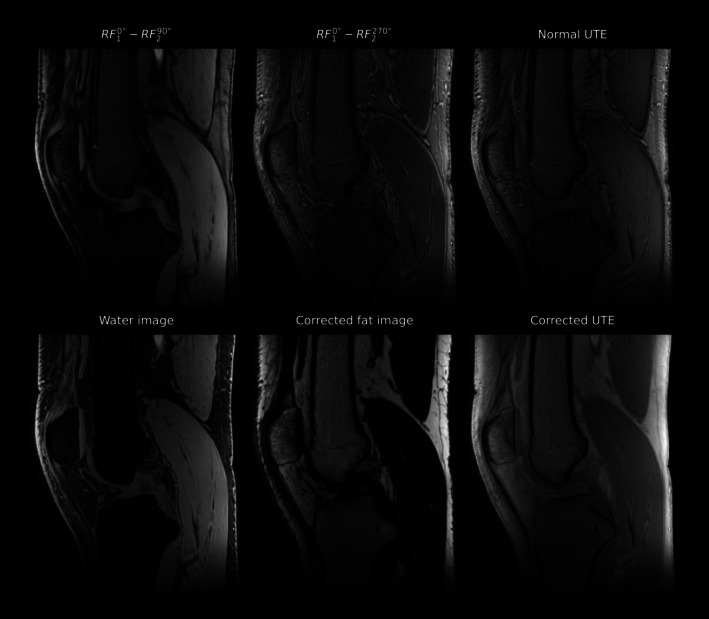
Central slice of a knee scan acquired at 7 T. The top row shows images obtained using the proposed binomial pulse configurations, alongside a conventional UTE scan employing a single pulse excitation. The bottom row displays the same slice after separation into fat and water images and the chemically shift‐corrected fat and water signals combined to produce a chemically shift‐corrected UTE image.

**FIGURE 7:**
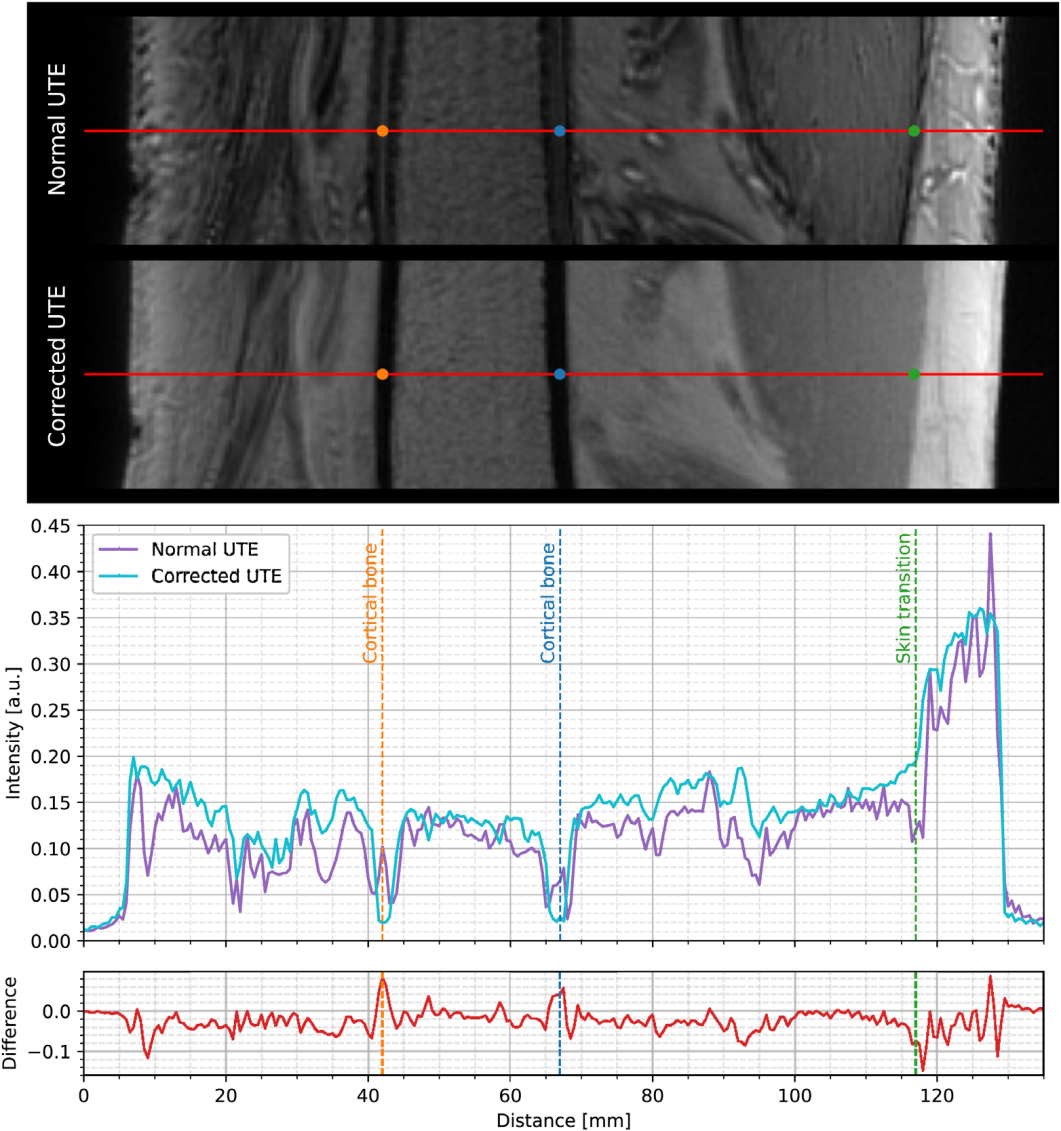
Horizontal profile position (red in top images) drawn through the femur in the sagittal 7 T slice depicted in Figure 6 and the corresponding line intensity profiles for the normal UTE (purple) and chemically shift‐corrected UTE (cyan) reconstructions. Reference positions for the cortical bone (orange and blue points) and posterior muscle to fat (green points) transitions drawn along the profile line are depicted as corresponding dashed vertical lines in the line profiles at the bottom. The difference between the profile line of the normal UTE and chemically shift‐corrected UTE is shown in the bottom.

High‐resolution knee imaging acquired at 7 T demonstrates a pronounced modification of image contrast when using the ${\mathrm{RF}}_{1}^{0{}^{\circ}}-{\mathrm{RF}}_{2}^{90{}^{\circ}}$ and ${\mathrm{RF}}_{1}^{0{}^{\circ}}-{\mathrm{RF}}_{2}^{270{}^{\circ}}$ pulse schemes (Figure 6, first row left and center image), indicating that the individual binomial scans alone provide limited image contrast and quality. The last image in the top row of Figure 6 further demonstrates that high‐resolution UTE imaging at high field strength is strongly affected by chemical shift artifacts when acquired without fat saturation/suppression. The bottom row of Figure 6 presents the results after applying the proposed fat–water separation and chemical shift correction, demonstrating robust fat–water decomposition across the entire image. The third image (bottom right) combines the corrected water and fat signals, yielding an image free from chemical shift‐induced blurring of the fat component. A corresponding horizontal profile line drawn through the femur within the same slice is shown in Figure 7 for both the conventional UTE and the chemically shift‐corrected UTE reconstruction. The latter shows clearly reduced tissue boundary artifacts at fat/water transitions by removing the spatial misregistration of fat, thereby eliminating fat signal bleeding and interference patterns at tissue interfaces and therefore improving tissue boundary delineation. The severity of spatial misregistration can be especially well observed in the artifactual cortical bone signal in the conventional UTE that is completely removed by the chemical shift correction.

**FIGURE 8:**
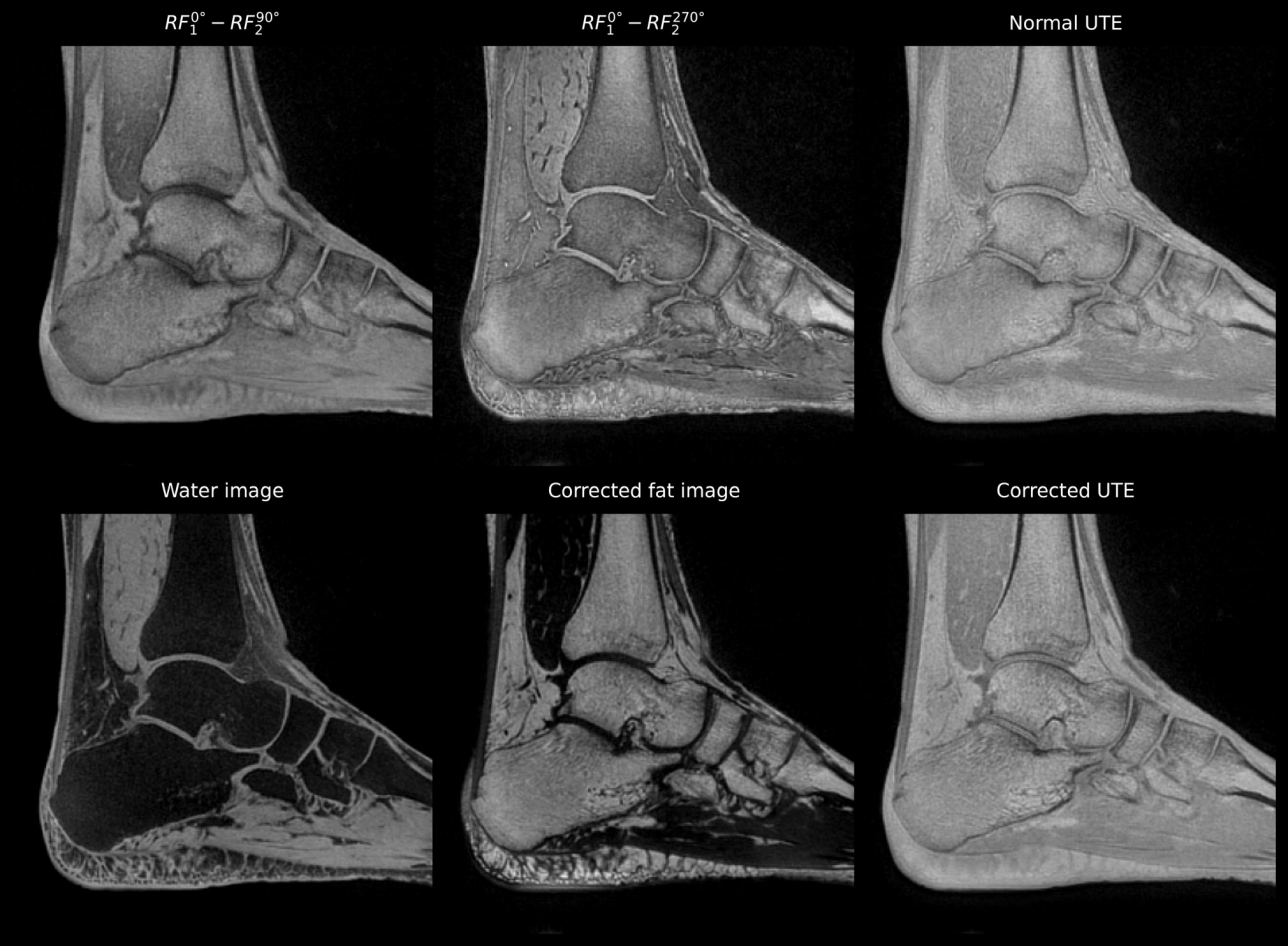
Sagittal slice of an ankle scan acquired at 3 T. The top row shows images obtained using the proposed binomial pulse configurations together with a conventional UTE acquisition exhibiting chemical shift artifacts. The bottom row presents the same slice after separation into fat and water images and the chemically shift‐corrected fat and water signals combined to produce a chemically shift‐corrected UTE image.

Figure 8 shows an exemplary in vivo acquisition of the ankle of a healthy volunteer at 3 T. The top row depicts the two acquisitions with the ${\mathrm{RF}}_{1}^{0{}^{\circ}}-{\mathrm{RF}}_{2}^{90{}^{\circ}}$ and ${\mathrm{RF}}_{1}^{0{}^{\circ}}-{\mathrm{RF}}_{2}^{270{}^{\circ}}$ pulse schemes, alongside a conventional UTE image acquired with a single pulse as reference. The reconstructed water and fat images (with applied chemical shift compensation), as well as the combined chemical‐shift free UTE are depicted in the bottom row. The chemical shift artifacts are less pronounced at 3 T compared to 7 T, since the acquisition bandwidth can still be set sufficiently high to reduce chemical shift‐induced blurring. Nevertheless, an improved boundary delineation can be perceived in the corrected UTE image compared with a conventional UTE image, especially as improved detail in the trabecular bone and at the muscle–fat interface. Furthermore, robust fat and water separation was achieved from the two ${\mathrm{RF}}_{1}^{0{}^{\circ}}-{\mathrm{RF}}_{2}^{90{}^{\circ}}$ and ${\mathrm{RF}}_{1}^{0{}^{\circ}}-{\mathrm{RF}}_{2}^{270{}^{\circ}}$ scans.

**FIGURE 9:**
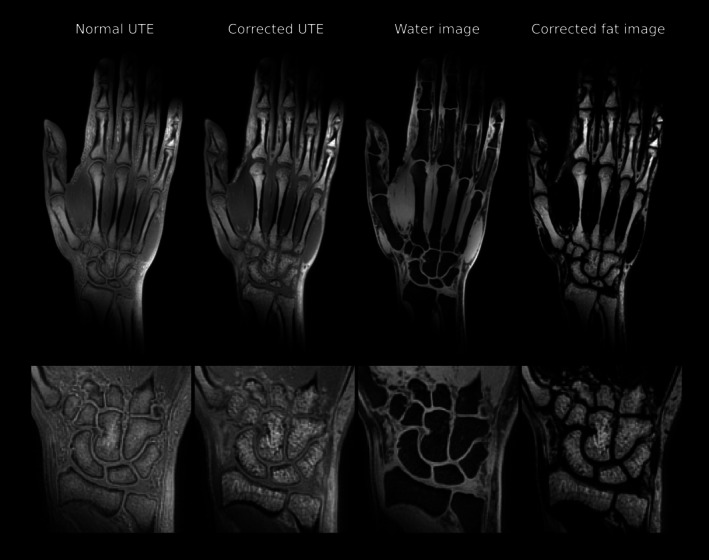
Coronal slice of a hand and wrist scan at 3 T in the top row, with a zoomed‐in view of the carpal bones in the bottom row. Shown from left to right are the conventional UTE acquisitions in comparison to the chemical shift‐corrected UTE and the separated water and fat images. The top view was generated using a curved surface reconstruction to simultaneously visualize the bones of all fingers in a single plane.

Shown in Figure 9 is a high‐resolution acquisition of the hand and wrist of a volunteer acquired at 3 T. To simultaneously visualize the bones of all fingers within a single plane, the top row displays a curved surface reconstruction. Consistent with the preceding results, the conventional UTE image exhibits chemical shift‐induced blurring that reduces the visibility of fine structures. In contrast, the corrected UTE image demonstrates an improved boundary delineation and detail definition. This is particularly evident in the magnified view of the carpal bones (bottom row), where the trabecular bone structure is more clearly delineated. Furthermore, robust water‐fat separation was achieved throughout the complex anatomy of the hand, allowing for a clear distinction between the lipid‐rich bone marrow and the surrounding soft tissue and muscular structures.

**FIGURE 10:**
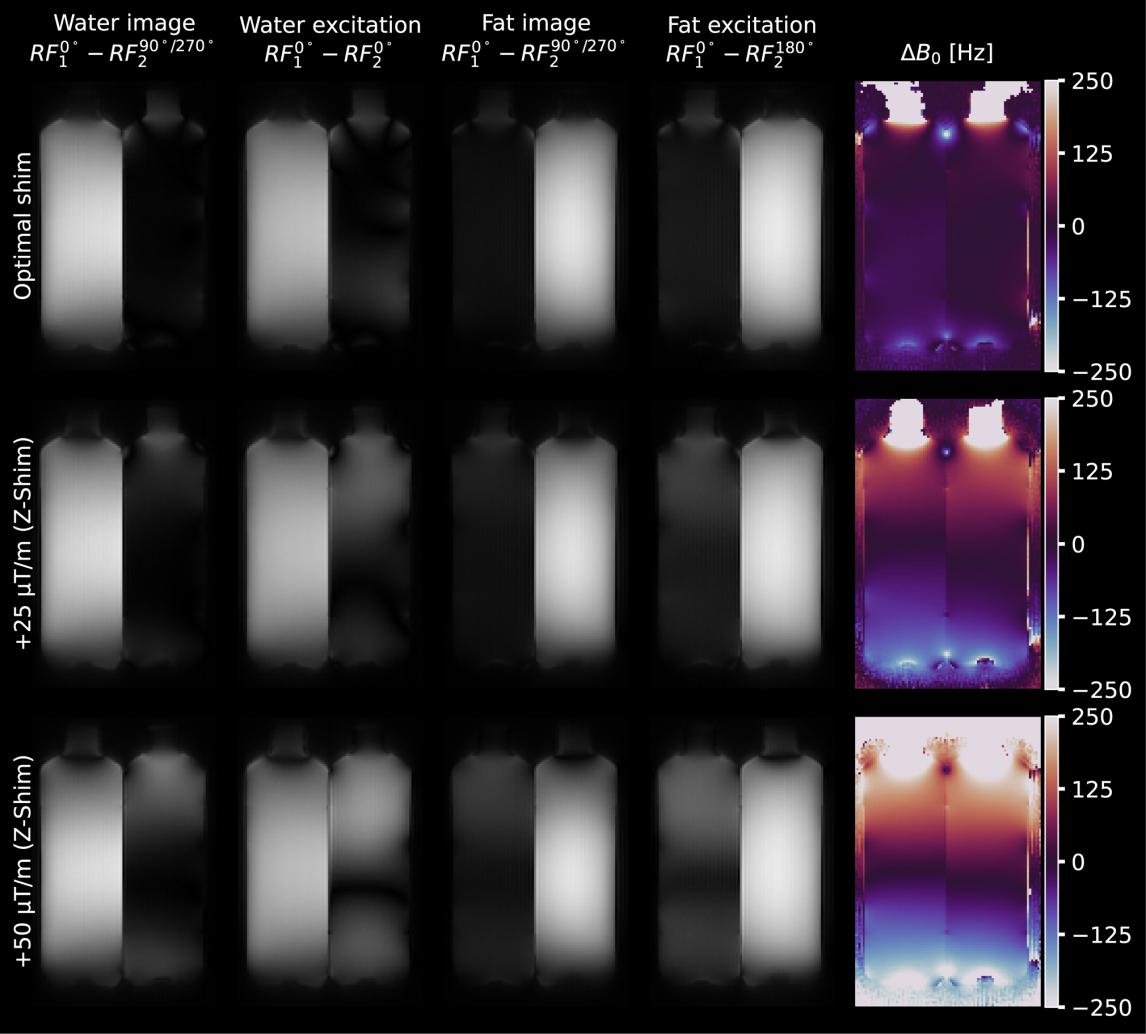
Comparison of fat‐water separation robustness against $B_{0}$ inhomogeneity in a phantom setup (left bottle: water; right bottle: vegetable oil). The rows correspond to optimal shim (top), and modified z‐shims of +25 μT/m (middle) and +50 μT/m (bottom). The columns display: (first) Water image reconstructed using the proposed method; (second) Standard ${\mathrm{RF}}_{1}^{0{}^{\circ}}-{\mathrm{RF}}_{2}^{0{}^{\circ}}$binomial water excitation; (third) Fat image using the proposed method; (fourth) Standard ${\mathrm{RF}}_{1}^{0{}^{\circ}}-{\mathrm{RF}}_{2}^{180{}^{\circ}}$ binomial fat excitation; and (fifth) measured ${\Delta B}_{0}$ maps.

The performance of the proposed method in the presence of $B_{0}$ inhomogeneities is illustrated in Figure 10 and compared to standard binomial excitation. Under optimal shim conditions (top row), both approaches provided effective spectral separation of the water and oil compartments, with noticeable signal leakage (incomplete suppression) in the oil compartment of the water‐selective excitation. Upon introducing linear field gradients to deliberately perturb *B*_*0*_ homogeneity, both methods exhibited signal nonuniformities corresponding to the increasing off‐resonance along the z‐direction. The conventional water ${\mathrm{RF}}_{1}^{0{}^{\circ}}-{\mathrm{RF}}_{2}^{0{}^{\circ}}$and fat ${\mathrm{RF}}_{1}^{0{}^{\circ}}-{\mathrm{RF}}_{2}^{180{}^{\circ}}$excitation schemes displayed characteristic banding artifacts as the field inhomogeneity increased. Similarly, the proposed method was not immune to these effects, showing signal intensity variations with increasing off‐resonance. However, the resulting artifacts were qualitatively less extensive and less severe than those observed with the standard reference binomial water‐ and fat‐selective excitation scheme.

## 5 Discussion

The primary objective of this work was to introduce a novel methodology for mitigating chemical shift artifacts and enabling robust fat‐water separation in UTE imaging. This was achieved by developing a modified binomial excitation scheme combined with chemical shift correction during reconstruction applied directly to the readout signal. As a result, the chemical shift‐corrected UTE images demonstrated significant improvements in image quality, particularly at high field strengths.

The derived equations and the experimental validation consider only a single‐peak fat signal as off‐resonant component. This differs from modern Dixon or IDEAL methods, which typically incorporate multi‐peak spectral fat models to enable accurate quantitative assessment of fat and water fractions [19, 20]. While our single‐peak assumption naturally limits the accuracy of quantitative fat fraction estimation, it is adequate for morphological fat‐water separation and for reducing chemical shift‐induced blurring. One specific consequence of this simplification is that olefinic (vinyl) protons, which resonate close to the water frequency, accumulate little phase during $t_{\mathrm{oop}}$ and are therefore assigned predominantly to the water image. The presented concept could potentially be extended to a multi‐peak spectral model by acquiring data with multiple different delay times $t_{\mathrm{oop}}$, though this would come at the expense of increased scan time. Additionally, by adjusting the delay time $t_{\mathrm{oop}}$ between the phase‐shifted binomial pulses, the presented concept should already be applicable to other chemically shifted components, such as, for example, silicone. Furthermore, due to the severity of chemical shift artifacts in high resolution or ultra‐high‐field UTE imaging, this work focused solely on 3D UTE sequences employing short rectangular pulses. Nevertheless, the presented concept should be broadly applicable to various pulses and readout schemes, including longer, slice‐selective sinc‐pulses in combination with Cartesian imaging readouts. For such implementations, however, extensive tweaking of the pulse duration and timing will be required to accommodate the respective slice selection gradients and longer pulses within $t_{\mathrm{oop}}$. Alternatively, a delay time corresponding to later out‐of‐phase conditions between fat and water (e.g., 540°) could be used, providing increased temporal flexibility to play out slice selection gradients.

A limitation of the proposed method is the prolonged acquisition time. Specifically, compared to a matching standard UTE acquisition, the water/fat separation requires two acquisitions where each TR is further extended by $t_{\mathrm{oop}}$. The TR penalty is, however, relatively small at high field strengths, as $t_{\mathrm{oop}}$ decreases linearly with field strength. For applications that require solely fat suppression, a single acquisition using an optimized spectral saturation pulse (e.g., a 1‐2‐1 binomial pulse) is generally more time‐efficient, as it increases the TR only by the saturation pulse duration rather than doubling the scan time. However, for applications that require separate water and fat images (i.e., explicit separation) or a non‐fat‐saturated contrast while mitigating chemical shift, conventional saturation approaches would likewise require two separate acquisitions. In this setting, the proposed method becomes advantageous because the 1‐1 excitation uses a shorter delay than a separate saturation pulse, enabling shorter minimum TR. This benefit is most pronounced for UTE acquisitions with short readouts, where the overhead of a second scan is balanced by the short TR. Conversely, for sequences with long or multiple readouts per excitation, the penalty of a second acquisition makes standard saturation techniques the more efficient choice. In comparison, classical multi‐echo Dixon‐based approaches also require an extended TR to acquire at least one additional echo. An advantage of performing two acquisitions with different pulse configurations but otherwise identical parameters is that, in contrast to Dixon based approaches, all data required for fat–water separation and chemical shift correction are acquired at the same echo‐time, ensuring identical *T*_2_* decay and susceptibility artifacts.

To calculate the required phase offsets $\Delta \phi_{W}$ and $\Delta \phi_{F}$ for fat–water separation, we neglected possible $T_{2}^{*}$ decay occurring during $t_{\mathrm{oop}}$. This simplification allowed all processing to be performed directly at the readout level, thereby enabling chemical shift correction. Alternatively, voxel‐wise fat and water separation in image space could be performed with the additional incorporation of a separately measured $T_{2}^{*}$ map for accurate estimation of $\Delta \phi_{W}$ and $\Delta \phi_{F}$. When neglecting $T_{2}^{*}$, it is important to note that the resulting error in $\Delta \phi_{F}$ is most likely relatively small, as the effective $T_{2}^{*}$ of fat is typically significantly longer than $t_{\mathrm{oop}}$[21]. Since $\Delta \phi_{F}$ is used to calculate the water signal, the resulting water image should be impacted very little by this approximation. The fat image, on the other hand, may exhibit imperfect separation in tissues with very short $T_{2}^{*}$, as various tissues—such as cortical bone [22], tendons and ligaments [23]—are characterized by $T_{2}^{*}$ values comparable to or even shorter than $t_{\mathrm{oop}}$. Therefore, the fat image may contain signal contamination from tissues that do not arise purely from fat but reflect imperfect separation. To address this in future applications targeting ultra‐short $T_{2}^{*}$ species with $T_{2}^{*}\ll t_{\mathrm{oop}}$, the method could be extended to a multi‐point excitation scheme by acquiring additional data with varying, shorter pulse spacings (e.g., fractions of $t_{\mathrm{oop}}$ and accordingly adjusted phase angles for the second pulse). It is also important to consider that both $t_{\mathrm{oop}}$ and $T_{2}^{*}$ depend on field strength [24, 25]. Hence, the influence of neglecting $T_{2}^{*}$ on $\Delta \phi_{W}$ will vary with field strength and tissue type. Additionally, the calculation of the phase offset $\Delta \phi_{F}$ using the approximated equations is only valid for small flip angles and short pulse durations. This limitation is generally not problematic for most UTE acquisitions, which typically employ short pulses and short TRs. However, it becomes an important consideration when using larger flip angles in combination with longer pulses, for example in variable flip angle‐based *T*_1_ mapping [26].

It is furthermore important to acknowledge that the accuracy of the fat‐water separation and chemical shift correction relies on precise knowledge of the fat‐water chemical shift and consistent magnetic field homogeneity. Phantom measurements confirmed that pronounced magnetic field inhomogeneities can compromise separation efficiency, leading to signal inhomogeneities and incomplete suppression of the undesired species. Crucially, the proposed method exhibited separation stability comparable to standard reference binomial water‐ and fat‐selective excitation schemes, with qualitatively reduced signal leakage in regions of high off‐resonance. This demonstrates that the sensitivity to $B_{0}$ inhomogeneities is inherent to the binomial excitation principle and is not exacerbated by the proposed modified pulse scheme for chemical shift separation and correction. While our in vivo experiments demonstrated robust performance even at 7 T, significant field inhomogeneities or deviations from the assumed chemical shift may reduce the effectiveness of the method. In this work, the delay time $t_{\mathrm{oop}}$, was manually determined from the peak separation of water and fat observed in the vendor's frequency adjustment display. Future work could explore the implementation of a rapid, automated calibration pre‐scan to precisely measure the fat‐water frequency difference, either globally or within a specific region of interest. This, in combination with field map‐based [27] correction techniques, could further improve robustness in anatomically challenging regions or in patients with metallic implants.

## 6 Conclusion

In this work, we presented a novel approach to address chemical shift artifacts and achieve robust fat‐water separation in UTE imaging. Building upon the principles of binomial excitation pulses, we developed a modified excitation scheme that enables the decomposition of distinct water and fat signals. Our proposed method not only effectively separates these components but also allows for correction of chemical shift directly at the readout level, resulting in substantially improved image quality, particularly at high field strengths, where chemical shift artifacts are more pronounced. Through theoretical derivations, Bloch simulations, and in vivo experiments at 3 T and 7 T, we demonstrated the efficacy and potential utility of the proposed technique for enhancing the diagnostic quality of UTE MRI.

## Funding

This work was supported by Deutsche Forschungsgemeinschaft (KR 4783/2‐1 and 576738170).

## Conflicts of Interest

Lumeng Cui and Stefan Sommer are employed by Siemens Healthineers.

## Data Availability

The data that support the findings of this study are available on request from the corresponding author. The data are not publicly available due to privacy or ethical restrictions.
